# Compressive Viscoelasticity of Freshly Excised Mouse Skin Is Dependent on Specimen Thickness, Strain Level and Rate

**DOI:** 10.1371/journal.pone.0120897

**Published:** 2015-03-24

**Authors:** Yuxiang Wang, Kara L. Marshall, Yoshichika Baba, Ellen A. Lumpkin, Gregory J. Gerling

**Affiliations:** 1 Department of Systems and Information Engineering, University of Virginia, 151 Engineers Way, Charlottesville, Virginia, 22903, United States of America; 2 Department of Mechanical and Aerospace Engineering, University of Virginia, 122 Engineers Way, Charlottesville, Virginia, 22903, United States of America; 3 Department of Biomedical Engineering, University of Virginia, 415 Lane Road, Charlottesville, Virginia, 22908, United States of America; 4 Department of Dermatology, Columbia University College of Physicians & Surgeons, 1150 St. Nicholas Ave., New York, New York, 10032, United States of America; 5 Department of Physiology & Cellular Biophysics, Columbia University College of Physicians & Surgeons, 1150 St. Nicholas Ave., New York, New York, 10032, United States of America; Queen Mary University of London, UNITED KINGDOM

## Abstract

Although the skin’s mechanical properties are well characterized in tension, little work has been done in compression. Here, the viscoelastic properties of a population of mouse skin specimens (139 samples from 36 mice, aged 5 to 34 weeks) were characterized upon varying specimen thickness, as well as strain level and rate. Over the population, we observed the skin’s viscoelasticity to be quite variable, yet found systematic correlation of residual stress ratio with skin thickness and strain, and of relaxation time constants with strain rates. In particular, as specimen thickness ranged from 211 to 671 μm, we observed significant variation in both quasi-linear viscoelasticity (QLV) parameters, the relaxation time constant (τ_1_ = 0.19 ± 0.10 s) and steady-state residual stress ratio (G_∞_ = 0.28 ± 0.13). Moreover, when τ_1_ was decoupled and fixed, we observed that G_∞_ positively correlated with skin thickness. Second, as steady-state stretch was increased (λ_∞_ from 0.22 to 0.81), we observed significant variation in both QLV parameters (τ_1_ = 0.26 ± 0.14 s, G_∞_ = 0.47 ± 0.17), and when τ_1_ was fixed, G_∞_ positively correlated with stretch level. Third, as strain rate was increased from 0.06 to 22.88 s^−1^, the median time constant τ_1_ varied from 1.90 to 0.31 s, and thereby negatively correlated with strain rate. These findings indicate that the natural range of specimen thickness, as well as experimental controls of compression level and rate, significantly influence measurements of skin viscoelasticity.

## Introduction

The skin plays a critical role in protecting the musculoskeletal system and internal organs and serves to detect external stimuli. The skin’s mechanical properties greatly impact how these functions are performed. Understanding these properties is essential for many applications, including functional tissue engineering [1]; however a full characterization of skin mechanical properties has not been accomplished due to its structural complexity. Skin consists of a multilayered epidermis and dermis [2] tied together by undulating interfaces embedded with pegged rete ridges. Each layer is different in both structure and function. For example, the outer stratum corneum of the epidermis is dry enucleated tissue that is stiffer the than remaining four layers of epidermis and serves as a physical barrier to the external environment. The dermis is made up of an extracellular matrix that includes collagen, elastin, and proteoglycans, among other components. Whereas the collagen and elastin fibers well account for the skin’s mechanical behavior under tensile loading [3,4], further work suggests the filler substance of proteoglycans between cells may dictate the skin’s behavior under compressive loading [5].

The skin’s mechanical properties, especially viscoelastic relaxation, have been studied routinely in tension [3,4,6–8] but much less in compression, where they are likely to differ significantly. In addition, despite prior efforts at sub-micron scales [9,10], few studies focus on macro-scale, bulk material measurements [11,12], which are useful in continuum methods such as finite element analysis.

One open question is to what extent individual differences impact the range of skin relaxation (e.g., time constants and residual stress ratios). For example, individuals display a wide range of variability in skin properties at different body sites and during aging [13,14]. While only single-specimen experiments have been performed in compression [11], multiple-specimen results from skin in tension shed some light on this question. For example, investigations with a twistometer indicate that human skin thickness decreases after about 20 years of age [15] and aging speeds up skin relaxation [16]. In mice, skin relaxation in tension also depends on animal age and body site [16,17]. Therefore, while we know both animal age and body site correlate with thickness [12], we do not understand how variability in thickness influences the relaxation of the skin under compression. The skin’s relaxation, and its variance between individuals, may impact somatosensory neural responses underlying the sense of touch [18], and thus is important for designing haptic devices to robustly and consistently deliver stimuli to the fingertip.

Beyond natural individual differences, biological material relaxation can be influenced by strain level and rate. Our understanding of such factors are vital to deciphering how we secure objects that are slipping from our grasp, for example [19]. Under tensile loading, Lanir has identified skin viscoelasticity to be strain-level dependent, where relaxation periods are elongated under larger strain [4,20]. Along the same lines, measurements of ankle ligaments indicate that the residual stress ratio decreases under larger strain [21]. Strain rates can significantly affect viscoelastic measurements as well. As shown for both articular cartilage [22] and human knee ligament [23], greater strain rates lead to greater peak forces.

In summary, the existing literature does not sufficiently describe the viscoelasticity of the skin, especially 1) in compression and 2) across a population of specimens with natural, individual differences, and 3) where strain level and rate can influence the results. The present study addresses these gaps in conducting compressive uniaxial tests on freshly excised mouse skin. Mouse skin was used because its thickness can be controlled through genetics, housing conditions and diet. The mouse is also the most widely used mammalian model system. To achieve different thicknesses, we sampled specimens from animals varying in age, hair cycle, body weight and skin site [12]. Specifically, the data were analyzed to determine if variability in skin thickness, as well as strain level and rate, contribute to variability in viscoelastic relaxation, as measured by relaxation time and steady-state residual stress ratio.

## Materials and Methods

### Overall

Uniaxial compression experiments on flat, cylindrically cut skin samples utilized controlled displacement ramped into the skin surface to collect time-force displacement data. For the purposes of analyzing the data, we generated material parameters of the quasi-linear viscoelasticity (QLV) model [24]. To decouple viscoelasticity from other factors such as material hyperelasticity and stimuli conditions, we obtain QLV parameters from a hyper-viscoelastic constitutive model and only examined its viscoelastic parameters, rather than comparing the force traces alone. The measured specimens were from 139 skin samples freshly excised from 36 mice, ranging 5.7–34.3 weeks in age, and from three skin sites on the hindlimb: distal (Distal), proximal on nerve trunk (NT), and off nerve trunk (OffNT). The three skin sites were selected due to differences in their thickness and underlying fascial structures [12].

Three independent variables were examined for their correlation with skin viscoelasticity: skin thickness (range from 211 to 671 μm, natural variation due to hair cycles over selected age), strain level (steady-state stretch *λ*_*∞*_ from about 0.2 to 0.8), and strain rate (median values of 0.06, 3.54 and 22.88 s^−1^). Strain level is defined as *ε* = |ln (*λ*)| = − ln (*λ*) in uniaxial compression, where *ε* denotes strain, *λ* denotes the stretch of material calculated from deformed thickness *l* divided by original thickness *l*_0_, $\lambda =\frac{l}{l_{0}}$. The strain level dependency was analyzed using stretch, which aligns with finite deformation theory [25] and negatively correlates with strain level in the case of compression. The rate of strain was defined as $\dot{\varepsilon }=\frac{d\varepsilon }{dt}$.

Finally, to validate that the viscoelastic parameters obtained in the skin compression experiments could be used to predict the behavior of the skin in a different context, we performed a secondary experiment with fresh mouse skin where we changed the stimulus, specimen size and different boundary condition. Finite element analysis was used to predict the results of this experiment.

### Ethics Statement

All animal use was conducted according to the National Institutes of Health *Guide for the Care and Use of Laboratory Animals* and was approved by the Institutional Animal Care and Use Committee of Columbia University (protocol AC-AAAC1561).

### Apparatus

**Fig 1:**
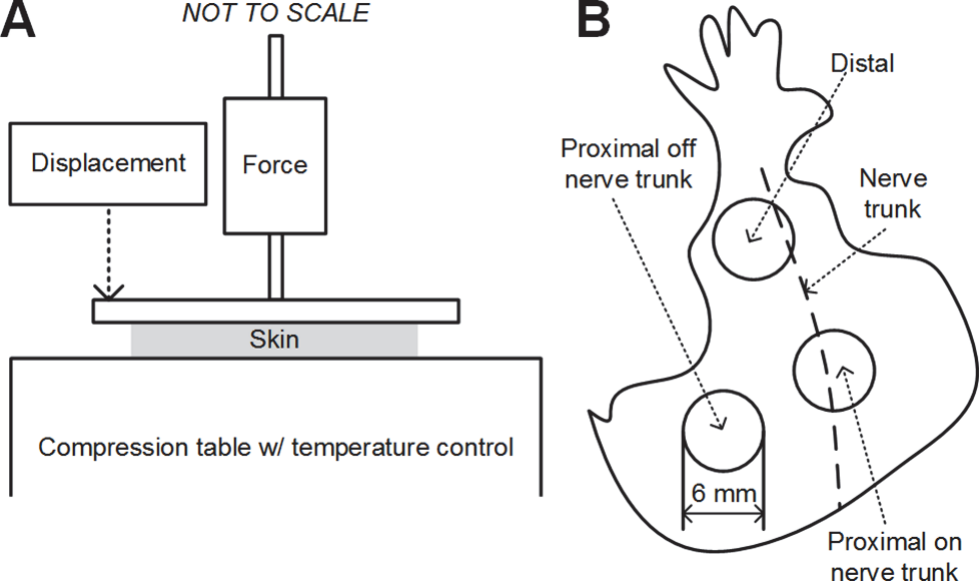
Schematics for the experiments. A: The experimental apparatus for uniaxial unconfied compression tests. B: Locations of the excised skin on mouse.

Compression tests were conducted on a custom-built test machine (Fig. 1A), described in depth elsewhere [12]. Briefly, the test machine’s components include a platen of aluminum (3 mm thick and 2.54 cm dia.) attached to a vertical load sled, which was driven by a motion controller (motion controller: Newport, Model ESP300, Mountain View, CA; linear stage: Newport, Model ILS100. Reaction force at the platen was measured by a loadcell (Honeywell, Miniature Model 31, Columbus, OH) with full capacity of 2.45 N mounted between the platen and vertical load sled, and its position was tracked by a laser displacement sensor (optoNCDT Model ILD 1402, Micro-Epsilon, Raleigh, NC) with a resolution of 1 μm. Both force and displacement were sampled at 1 kHz. The platen compressed the skin specimens against a rigid platform parallel to its surface, with sides of the cylindrical skin unconfined. The apparatus was equipped with a closed-loop control system integrated to maintain temperature of 32 degrees Celsius, consistent with prior works [26], using a BASIC Stamp microcontroller (Parallax Inc., Rocklin, CA) and associated electronic transistors and heating elements.

### Animals and dissection

The animal preparation and dissection protocol has been described previously [12]. Skin samples were obtained using a 6-mm diameter punch (Acuderm Inc., Ft. Lauderdale, FL) after skin specimens were dissected from the mouse hindlimb. Three sampling sites (Fig. 1B) were chosen at distal end of the hindlimb (Distal), and the proximal end of the hindlimb both off (OffNT) and on (NT) the saphenous nerve trunk because these sites appear to be categorically differentiable in terms of thickness. Specimens were harvested from a total of 36 mice, at ages ranging from 5.7 weeks to 34.3 weeks and body weights ranging from 15.9 grams to 61.4 grams. A total of 139 skin samples including 46 from Distal, 46 from OffNT and 47 from NT areas were harvested.

### Skin test procedure

For all specimens, we first set the starting position of the platen to ensure it was positioned above the flat skin surface by observing the reaction force. Next, displacement-controlled compression was applied with a ramp-up phase at a velocity to achieve the desired strain rate, a hold phase at the maximum load position for 6 seconds (note that only the first 5 seconds were used in analysis to avoid analyzing the ramp-off response), and an unloading phase of the same rate as the ramp-up. Multiple repetitions of same loading protocol were applied to the specimen, where the 6th run for each skin specimen was analyzed and the first 5 repetitions were used as pre-conditioning to minimize the variance due to stress history [27].

**Fig 2:**
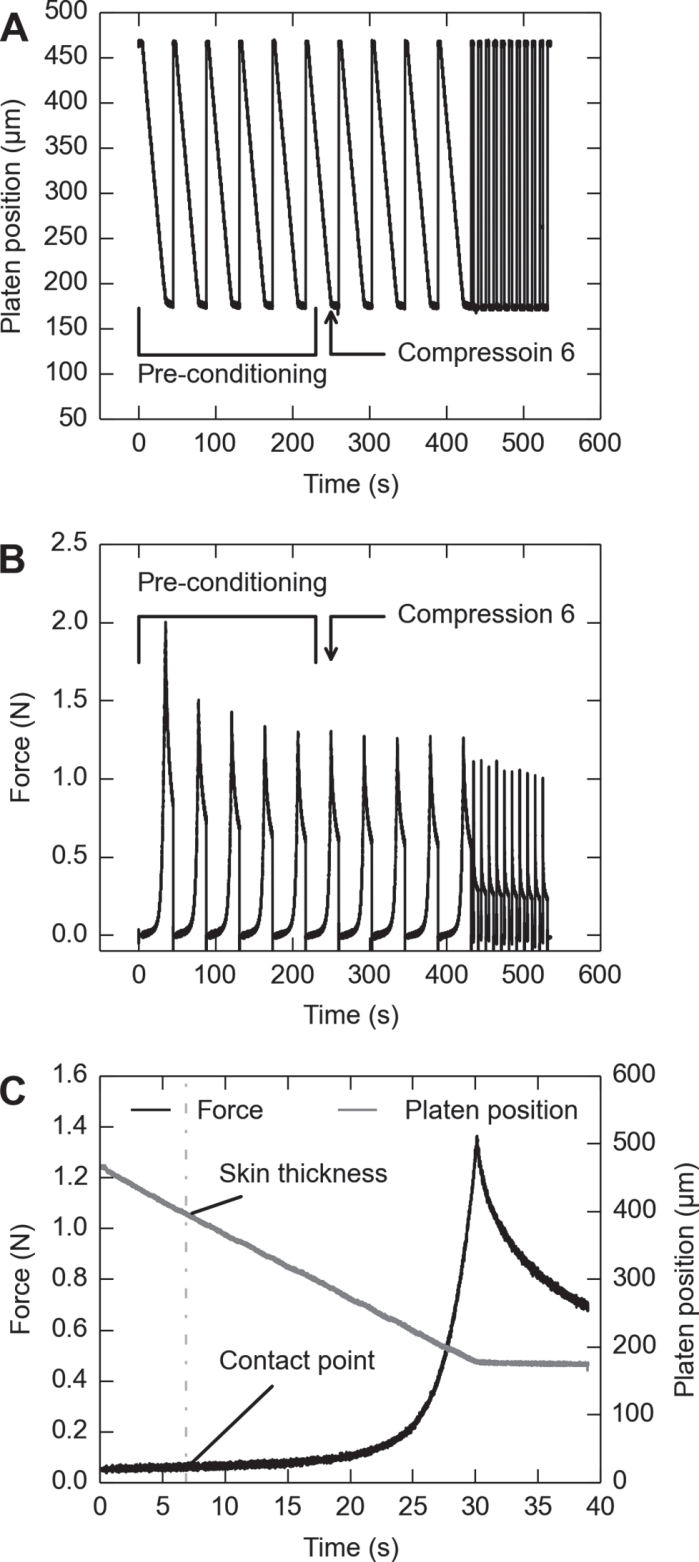
Example run of the compressive test procedure for one skin specimen when varying strain rates. A: Position of the compression platen over time, as measured by its distance from the fixed platform. B: Reaction force at the compression platen. C: Magnified view of reaction force and platen position for Compression 6, demonstrating that “skin thickness” was defined by “contact point” as determined from the force trace. The platen was moved into the skin with an acceleration of 0.06 s^−1^ for each of the first 10 repetitions. Then, 10 additional compressions were performed at 22.88 s^−1^. The 6th compression was analyzed in each sequence of 10 compressions.

Fig. 2 demonstrates a typical experimental procedure, where strain rates are varied. Synthetic interstitial fluid (SIF) [28] was added via eye dropper to prevent drying of the skin. The reaction force at the platen was measured by a loadcell and platen position measured by a laser displacement sensor. The recorded force trace was then used to determine the point of contact (Fig. 2C). A “light-contact point” of the platen to the specimen surface was determined at the moment when reaction force on the platen exceeded 0.1 N. After that, the “contact point” was defined as distance from the platen to the rigid table at the “light-contact point”, timed by a correction coefficient of 1.3. Then, the specimen thickness (*l*_0_) and deformed thickness (*l*) was each defined as the distance from the platen to the rigid table at and after the “contact point”.

**Fig 3:**
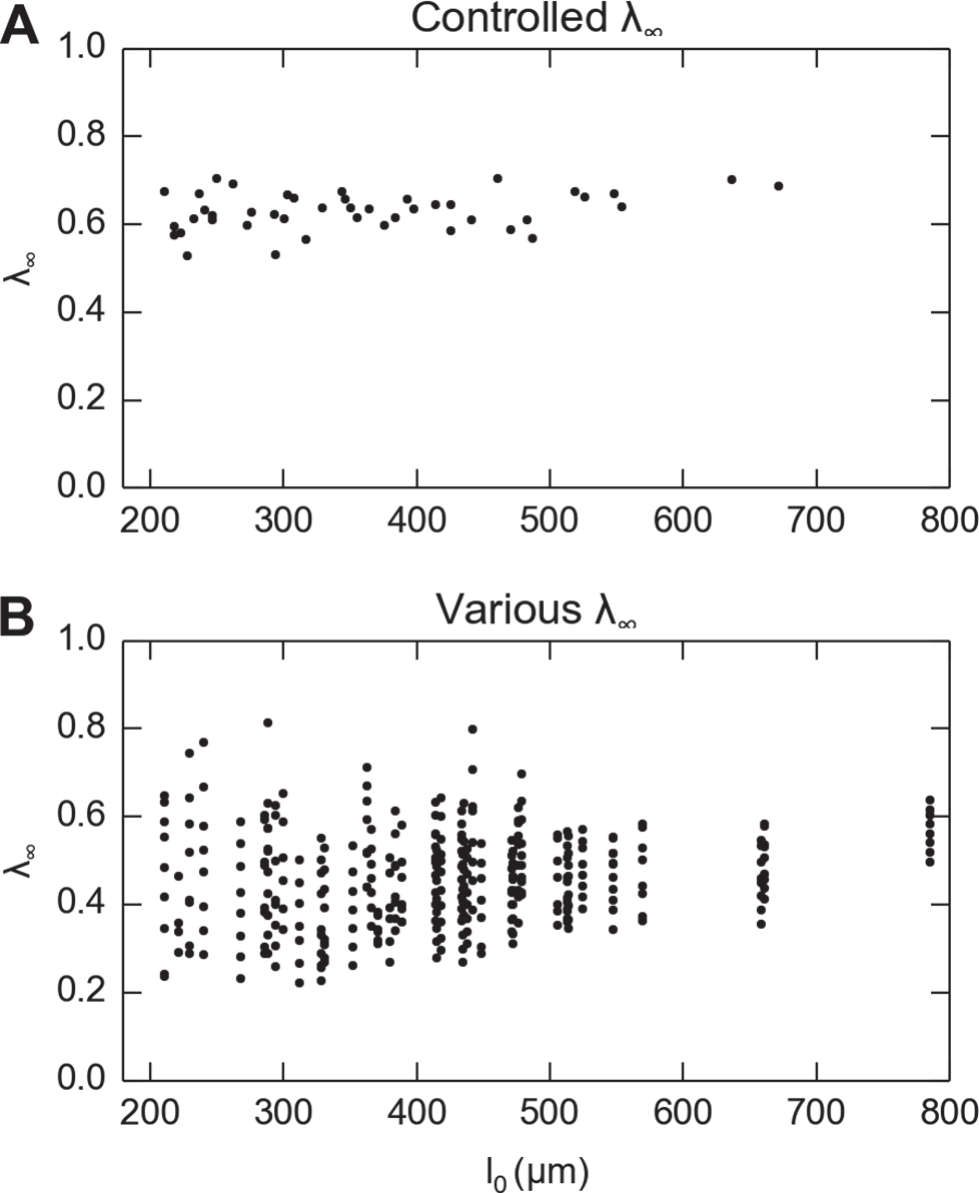
There is no correlation between the measured steady-state stretch (*λ*_*∞*_) and thickness. A: Skin thickness naturally varies between about 200 and 800 μm when a single, consistent stretch level of about 0.6 is delivered to each specimen in the first experiment. B: Skin thickness naturally varies when multiple stretch levels (*λ*_*∞*_ from about 0.2 to 0.8) are delivered to each of the skin specimens.

Three different experimental paradigms were used to elucidate viscoelastic properties of multiple skin samples under compression. First, we measured skin under the same level of strain from 44 skin samples of naturally varying thickness. Maximum indentation depths were determined by manually searching for an instantaneous reaction force around 2 N, which is the approximate magnitude to generate a level of stretch of 0.6, similar to indentation in neurophysiological studies [26]. The velocity of the compression platen was 1 mm/s to achieve a strain rate of about 3.54 s^−1^. Second, we applied similar procedures to 41 skin samples, but linearly varied steady-state stretch for each specimen. The minimum stretch level was set when the responsive force recorded at the loadcell was above zero, determined by its magnitude being one standard deviation above background noise, and the maximum stretch level was set when the maximum responsive force reached 2.45 N. Any force above this level was avoided to prevent damage to the skin or instrumentation. Fig. 3 demonstrates the difference in stretch level between the first and second experimental paradigms. In the third experiment, strain rate was varied. Two rates (medians are 0.06 and 22.88 s^−1^) were applied to 54 specimens, and the data were analyzed together with the first experiment (3.54 s^−1^) to constitute three strain rates at different orders of magnitude. The low strain rate ($\dot{\varepsilon }=0.06\;{\text{s}}^{-1}$) is comparable to activities with long relaxation times, such as standing and lying in bed, where one can still perceive the mattress even after several minutes. The medium strain rate ($\dot{\varepsilon }=3.54\;{\text{s}}^{-1}$) corresponds to typical light-touch activities, such as typing on a keyboard. The high strain rate ($\dot{\varepsilon }=22.88\;{\text{s}}^{-1}$) corresponds to impact loading, which one perceives to avoid imminent danger. The three strain rates correspond to 0.01, 1 mm/s and the fastest moving velocity of our test machine.

### Constitutive laws

The QLV model [24] was used here to fit the data to be analyzed. Given that our test was uniaxial, we only considered the one-dimensional situation.

The QLV model utilizes the stretch-time curve to calculate the response stress, shown as

$$\sigma \left(t\right)=\overset{t}{\underset{-\infty }{\int }}\left(\overset{n}{\underset{i=1}{\sum }}G_{i}e^{-\frac{t-t'}{\tau_{i}}}+G_{\infty }\right)\frac{\partial \sigma_{e}\left(\lambda \right)}{\partial \lambda }\frac{\partial \lambda \left(t^{\prime }\right)}{\partial t^{\prime }}dt'\;.$$ (1)

For more details of the constitutive laws please refer to Appendix I.

### Fitting experimental data to constitutive model

**Fig 4:**
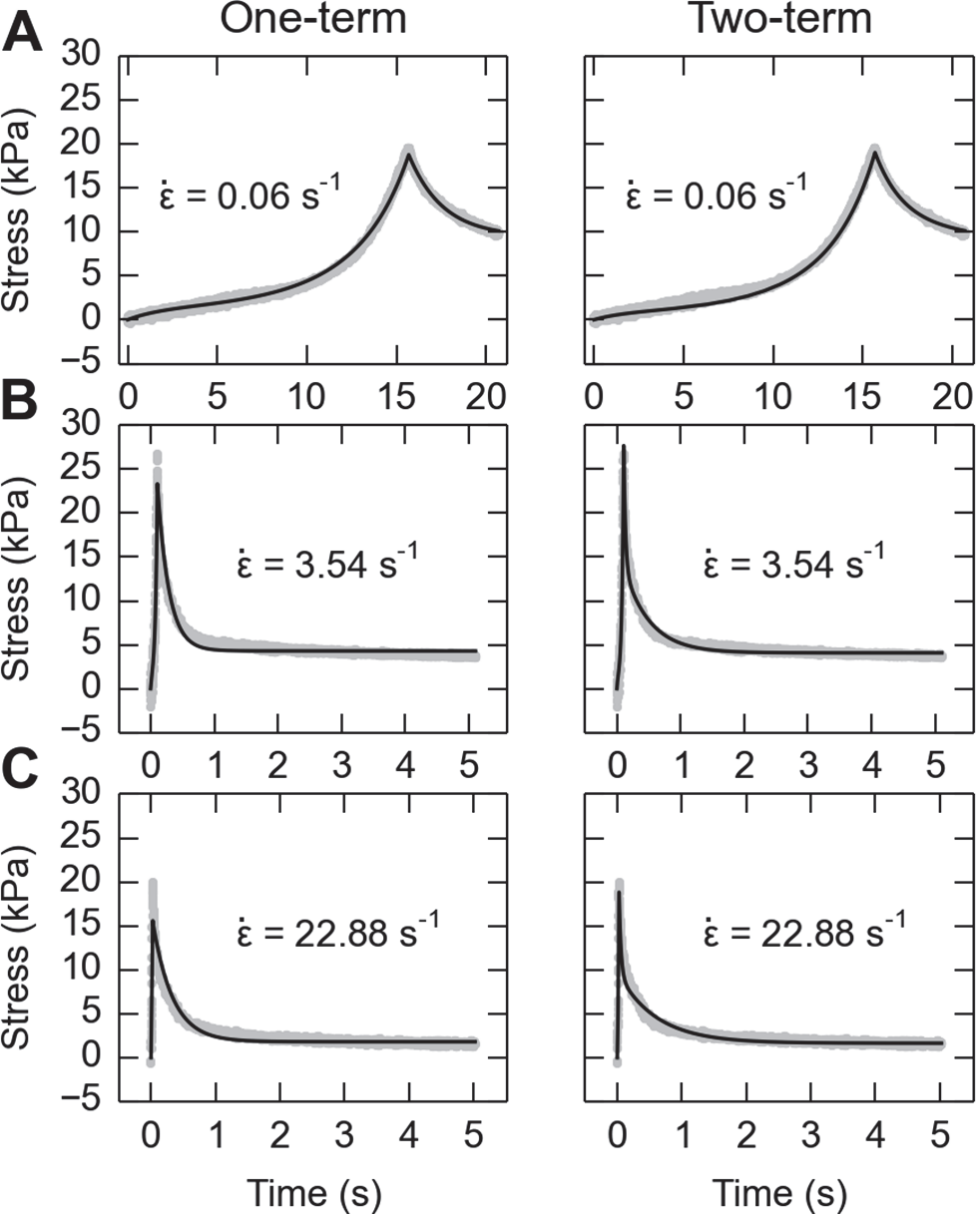
Example fit of stress over time by one-term (left column) and two-term (right column) QLV models at three strain rates (rows A: 0.06 s^−1^; B: 4.29 s^−1^; C: 35.34 s^−1^). Black line shows the modeled prediction, and gray data points show the experimental data. The average weighted *R* ^2^ value for the one-term case for the three strain-rates is 0.86 while the *R* ^2^ value using the two-term case is 0.93. Therefore, the tradeoff is that the number of free parameters increased from 1 to 2, versus attaining a slight improvement in the fitting, and for this reason we chose the one-term case.

To attain the parameters of the constitutive model, we fit the model to the stress-stretch-time curves calculated from the experimental data. The stretch value was calculated by dividing deformed thickness *l* over original thickness *l*_0_, i.e. $\lambda =\frac{l}{l_{0}}$ (l, *l*_0_ defined in Section 2.4). Recorded experimental force data were converted to stress values by dividing force over area, calculated from Equation (7), with the assumption that the volume of the specimen remained constant because it is nearly incompressible [24]:

$$A=\frac{A_{0}}{\lambda }\;,$$ (2)

where *A*_0_ is the area of the punch, a 6-mm diameter circle. For the detailed numerical algorithm used for fitting, please refer to the Appendix I. QLV model parameters were then adjusted to fit to the stress-time and stretch-time measurements (Fig. 4) taking the number of terms *n* = 1 and *n* = 2.

The stress and stretch history over the whole time window, including phases of both the ramp (from contact to peak stress) and hold (from peak stress to 5 seconds after), were used in the fitting to account for relaxation during loading, similar to Laksari et al. [29]. Because the number of data points in the hold phase were much greater than that in ramp phase, we used an *R* ^2^ value as the equally-weighted sum between the *R* ^2^ from fit of the ramp phase and hold phase. For each specimen, the weighted *R* ^2^ was maximized through a constrained nonlinear optimization (fmincon, in the MATLAB Optimization Toolbox) using the SQP algorithm. The reduced chi-square value ($\chi_{red}^{2}$) and the residual standard deviations (*σ*_*res*_) were also checked to assure the quality of fit.

Then, fitting of both one- and two-term models was performed in two steps:

1. We fit the experimental data with all free viscoelastic parameters (2 and 4 for one- and two-term models, respectively) and all free hyperelastic parameters (2 for each model), with manually chosen initial values for the optimization algorithm.
2. We fixed all time constant parameters (*τ*) and initial shear modulus (*μ*) to median values found in Step 1 between specimens tested under same strain rate. For the initial values for free parameters, corresponding median values found in Step 1 were used.

By fixing certain parameters in Step (2), the total number of free viscoelastic parameters were reduced to 1 for one-term (*G*_*∞*_) and 2 for two-term models (*G*_1_, *G*_*∞*_) respectively, and also included only 1 hyperelastic parameter (*α*) in each. For analysis of the distribution of time constants (*τ*), fitting results from Step 1 were used, and for that of stress ratios (*G*) results from Step 2 were used.

The fitting of QLV model to experiment data for each skin specimen was performed and results were listed in Table 1, which shows high *R* ^2^ values, $\chi_{res}^{2}$ close to 1 and low *σ*_*res*_ (< 1 kPa, compared to peak stress of about 50 kPa in the 2^nd^ experiment), indicating a good fit. Data in Table 1 and Fig. 4 reveal the trade-off for increasing the number of free parameters from 1 to 2 was attaining only a small improvement in fit. Thus, we decided to use the one-term model so that comparisons between specimens were easier with only a single free parameter. More importantly, by strictly controlling the number of free parameters, we minimized the non-unicity of the fitting.

**Table 1:** Median parameters from model fits to data for all experimental conditions.

Experiment	$\dot{\varepsilon }\;\left({\text{s}}^{\text{-1}}\right)$	Model	Material parameters							Goodness of fit		
			*τ*_1_(s)	*τ*_2_(s)	*G*_1_	*G*_2_	*G*_∞_	*μ*(kPa)	*α*	*R* ^2^	$\chi_{red}^{2}$	*σ_res_*(kPa)
First	3.54	One-term	0.180	-	0.748	-	0.252	6.422	10.703	0.870	1.001	0.448
		Two-term	0.028	0.410	0.631	0.189	0.143	7.958	12.683	0.910	1.001	0.325
Second	1.47	One-term	0.236	-	0.548	-	0.452	7.189	7.924	0.921	1.001	0.904
		Two-term	0.092	1.111	0.482	0.110	0.351	6.354	8.787	0.958	1.002	0.735
Third	0.06	One-term	1.900	-	0.684	-	0.316	7.162	6.511	0.974	1.328	0.456
		Two-term	1.569	50.895	0.612	0.368	0.000	5.453	7.573	0.974	1.471	0.475
	22.88	One-term	0.310	-	0.666	-	0.334	4.057	3.447	0.816	1.004	0.381
		Two-term	0.030	0.599	0.608	0.212	0.195	3.973	6.624	0.951	1.001	0.175

## Results

The parameters returned by fitting the one-term QLV model revealed that skin viscoelasticity is highly variable between specimens, yet correlates with the three independent variables. Specifically, the residual stress ratio *G*_*∞*_ positively correlates with skin thickness and stretch level, and the time constant *τ*_1_ negatively correlates with strain rate.

### Large variability between specimens

**Fig 5:**
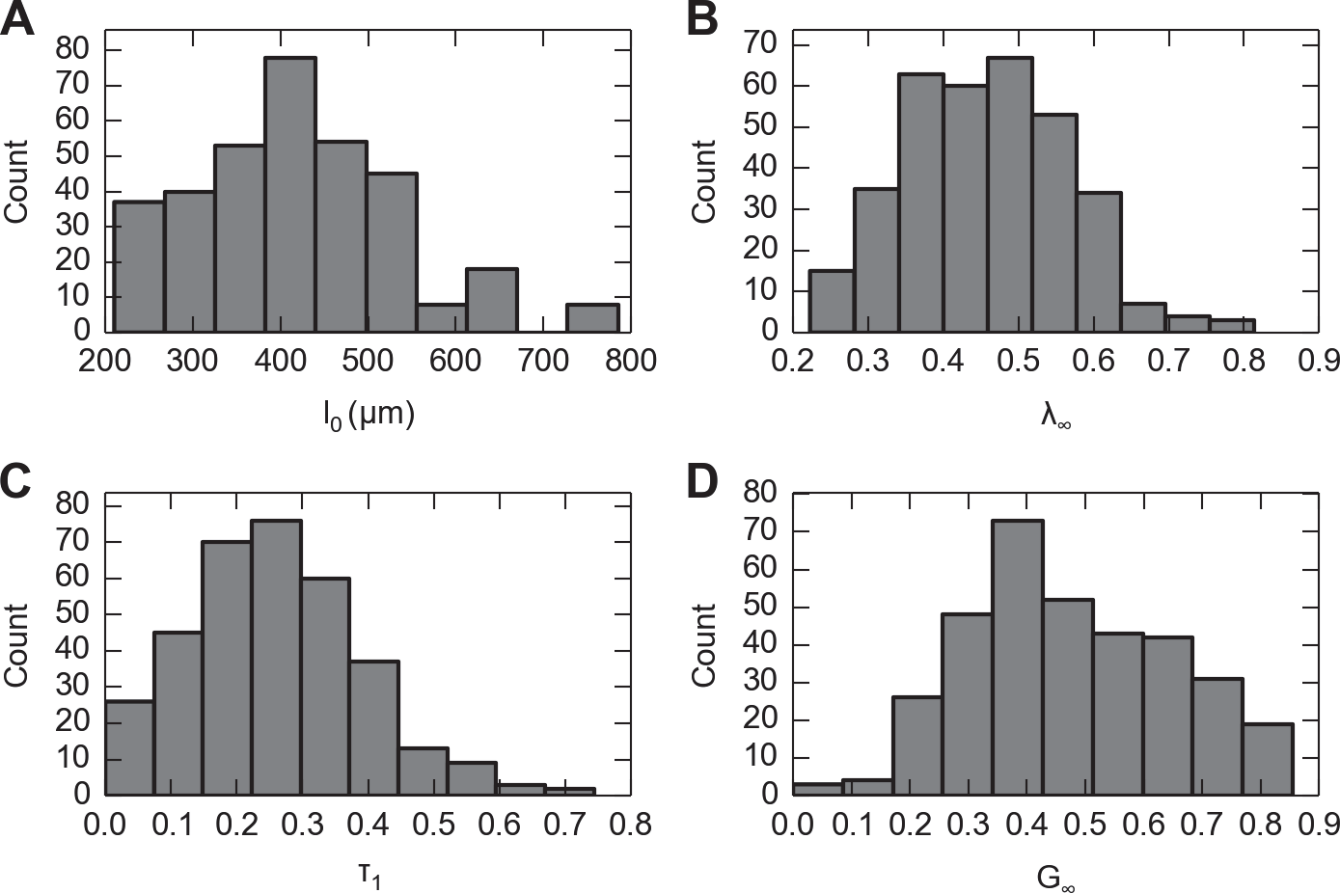
Data from 341 experimental runs (*n* = 41 specimens) each stimulated an average of eight stretch levels. Distributions are shown of A: skin thickness measurements; B: steady-state skin stretches (*λ*_*∞*_) applied; C: time constants from fitting stress versus time to the one-term QLV model; D: the steady-state residual stress ratio *G*_*∞*_. Note that each of the four variables exhibits high variability.

Large variability was observed from all three independent variables. First, as between specimen thickness changed from 211 to 671 μm, we observed significant variation in both QLV parameters: the relaxation time constant (*τ*_1_ = 0.19 ± 0.10 s) and the steady-state residual stress ratio (*G*_*∞*_ = 0.28 ± 0.13). Second, as skin thickness naturally varied (Fig. 5A) and steady-state stretch was increased (*λ*_*∞*_ from 0.22 to 0.81, Fig. 5B), we observed significant variation in both QLV parameters (*τ*_1_ = 0.26 ± 0.14 s, Fig. 5C; *G*_∞_ = 0.47 ± 0.17, Fig. 5D). Third, as strain rate was increased from 0.06 to 22.88 s^−1^, the median time constant *τ*_1_ varied from 1.90 to 0.31 s.

### Positive correlation between thickness and residual stress ratio

**Fig 6:**
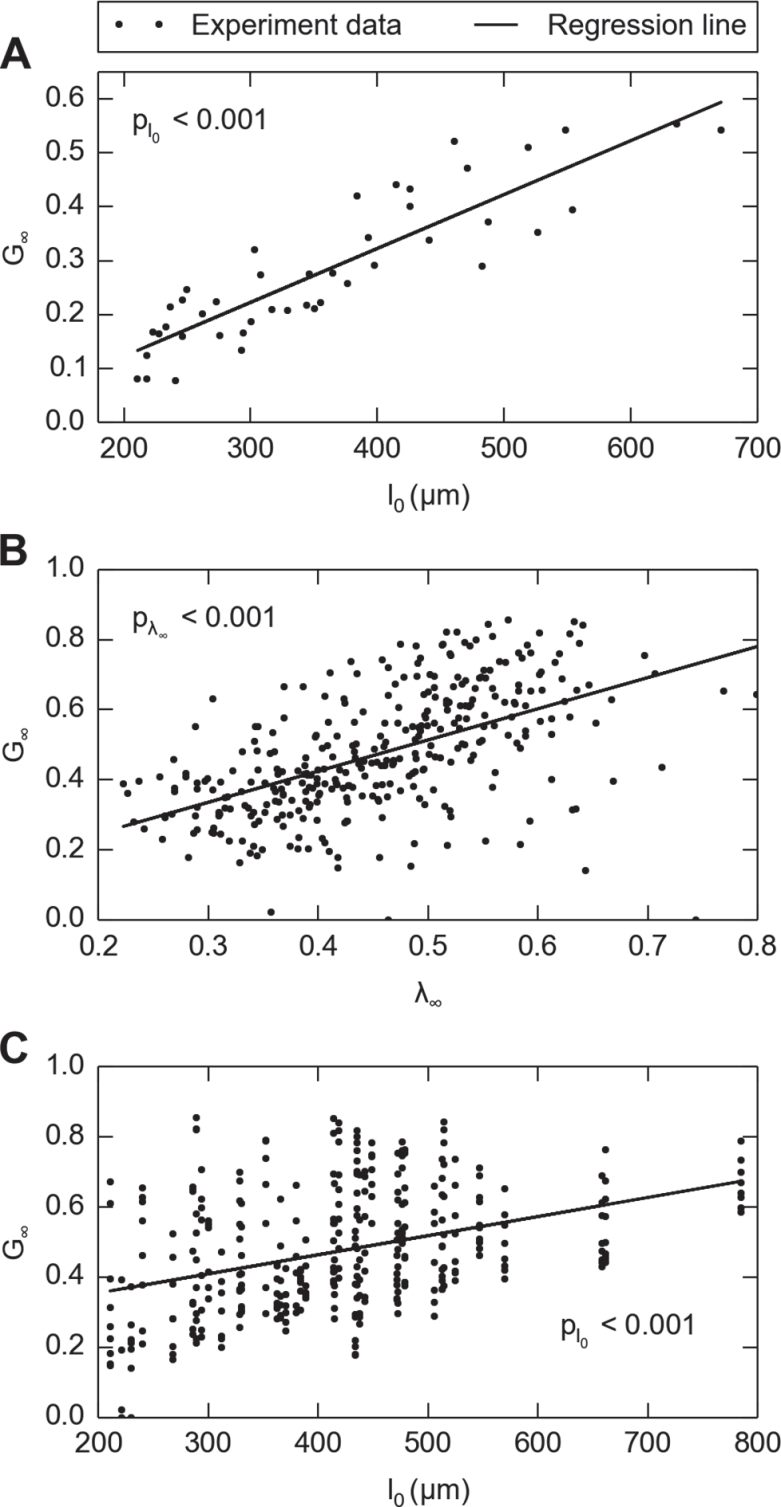
Correlations between skin thickness/stretch level and residual stress ratio (*G*_*∞*_). A: In the first experiment where only thickness varied, the steady-state residual stress ratio (*G*_*∞*_) correlates with increasing skin thickness, *n* = 44. Linear regression (solid line) with residual stress ratio *G*_*∞*_ as the dependent variable was performed, which returns *p* < 0.001 for independent variable thickness *l*_0_, and *G*_*∞*_ = 9.997 × 10^−4^ μm^−1^ ⋅ *l*_0_ + 0.077. In the second experiment where both thickness and strain level varied, the residual stress ratio (*G*_*∞*_) correlates with both B: stretch and C: skin thickness. Note that the two correlations are independent from each other because there is no correlation between stretch and skin thickness. Multilinear regression with residual stress ratio *G*_*∞*_ was also performed, which returns *p* < 0.001 for independent variable stretch *λ*_*∞*_, *p* < 0.001 for independent variable thickness *l*_0_, and *G*_*∞*_ = 0.810 ⋅ *λ*_*∞*_ + 4.25 × 10^−4^ μm^−1^ ⋅ *l*_0_–0.074. Note that in B and C, solid lines are single-linear regressions for residual stress ratio with respect to stretch and thickness respectively.

In the first experiment where the skin thickness naturally varied, residual stress ratio *G*_*∞*_ positively correlated with skin thickness, with Pearson correlation coefficient of 0.883 (Fig. 6A). Linear regression with residual stress ratio *G*_*∞*_ as a dependent variable was performed, which returned *p* < 0.001 for independent variable thickness *l*_0_, and *G*_*∞*_ = 9.997 × 10^−4^ μm^−1^ ⋅ *l*_0_ + 0.077.

### Strain-level dependency

In the second experiment where the change in stretch delivered accompanied skin thickness variation, the residual stress ratio *G*_*∞*_ was found to positively correlate with skin thickness (Fig. 6B), and moreover, also positively correlated with stretch level (Fig. 6C). Multilinear regression with residual stress ratio *G*_*∞*_ was also performed, which returns *p* < 0.001 for independent variable stretch *λ*_*∞*_, *p* < 0.001 for independent variable thickness *l*_0_, and *G*_*∞*_ = 0.810 ⋅ *λ*_*∞*_ + 4.25 × 10^−4^ μm^−1^ ⋅ *l*_0_ − 0.074, indicating that both skin thickness and stretch level are positively correlated with residual stress ratio and thus contribute to variability.

### Strain-rate dependency

**Fig 7:**
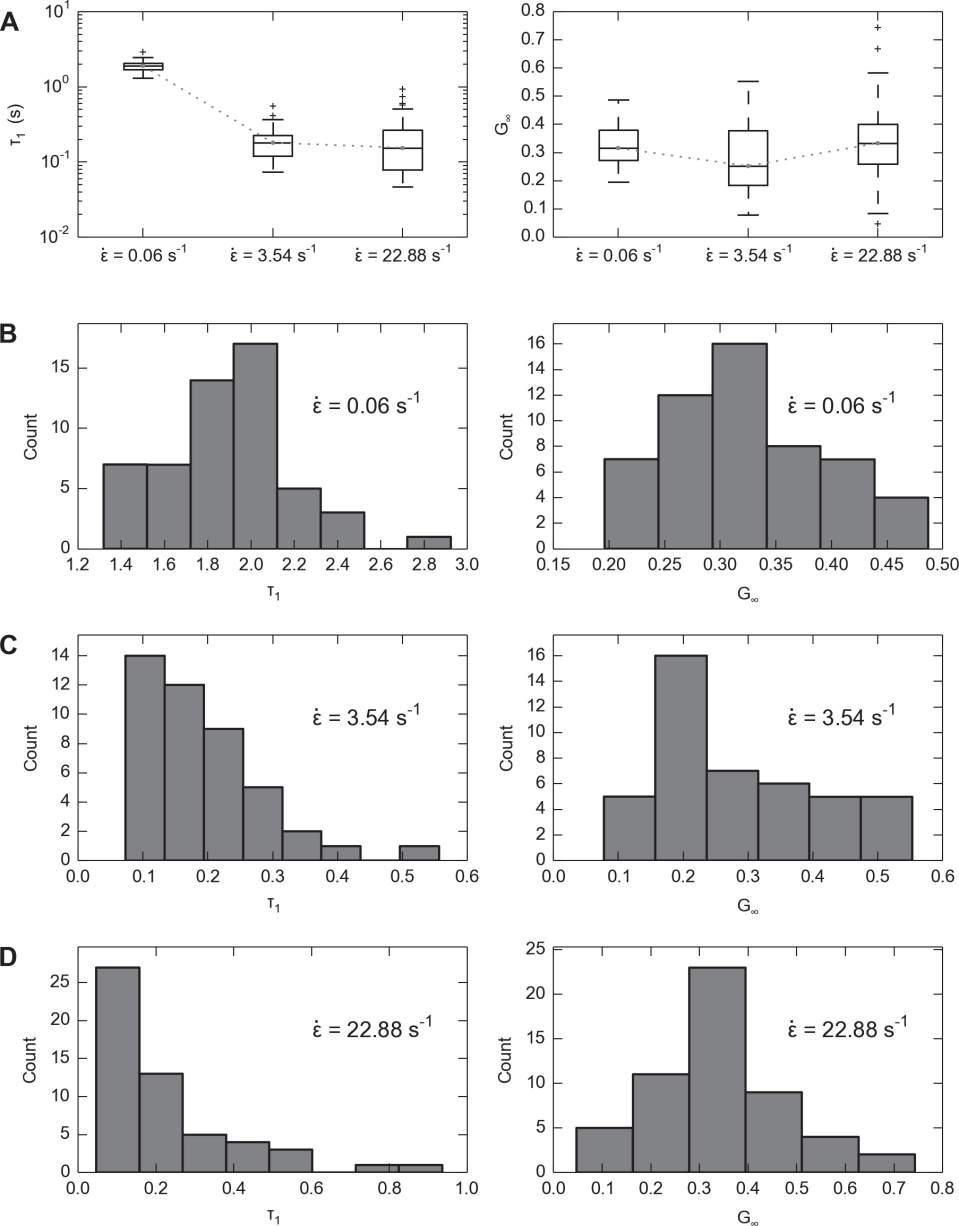
Values of time constant (*τ*_1_) and residual stress ratio (*G*_*∞*_) at three strain rates ($\dot{\varepsilon }$). A: Overall, the time constants are significantly smaller under faster strain rates, while there is no systematic trend in the change of residual stress ratio. The boxes range from the lower quartile to upper quartile, the centerlines denote the medians, the whiskers denote extreme values and crosses denote outliers. B-D: Detailed views of the distributions of time constants and residual stress ratios from all data points at B: strain rate 0.06 s^−1^, n = 54; C: strain rate 3.54 s^−1^, n = 44; D: strain rate 22.88 s^−1^, n = 54.

In the third experiment, the strain rate largely varied (median $\dot{\varepsilon }=0.06$, 22.88) and combined with data from first experiment (median $\dot{\varepsilon }=3.54$), we found that the same magnitude of relaxation takes place at significantly shorter time constants at higher strain rates (Fig. 7A). Linear regression was performed with time constant *τ*_1_ as the dependent variable and strain rate $\dot{\varepsilon }$ as the independent variable, which yielded a significantly negative correlation (*p* < 0.001) between the strain rate and time constant. Using the same regression but replacing the dependent variable from time constant *τ*_1_ with residual stress ratio *G*_*∞*_, we found that the strain rate did not significantly affect residual stress ratio (*p* = 0.988 > 0.05). With a closer examination of the distribution of time constants and residual stress ratios (Fig. 7B-D), we noticed that the distributions of time constants notably skewed to the left as strain rate increased, whereas the distribution of residual stress ratios did not show systematic changes.

## Discussion

This work shows, for the first time with mouse skin under compression, that skin’s viscoelasticity is highly variable (relaxation time constant *τ*_1_ = 0.19 ± 0.10 s and steady-state residual stress ratio *G*_*∞*_ = 0.28 ± 0.13) among a population of skin specimens (*n* = 139). However, we found systematic correlation in three cases: 1) the residual stress ratio *G*_*∞*_ positively correlates with skin thickness (*p* < 0.001), 2) residual stress ratio positively correlates with stretch level (*p* < 0.001), in other words, negatively correlates with strain level and 3) the time constant *τ*_1_ negatively correlates strain rate (*p* < 0.001). Overall, these findings shed light on the natural range of between-specimen variance under compression, and reveal how experimental controls of strain level and rate can influence measurement of the same specimen.

A small, secondary experiment with fresh mouse skin was performed to validate that the viscoelastic parameters obtained in the skin compression experiments could be used to predict the behavior of the skin in a different context. In particular, using the viscoelastic parameters obtained with the flat plate, we sought to predict the force relaxation of a 1.5 mm probe indented into a skin specimen of different cut-out size (8 mm as opposed to 6 mm), for two indentation depths. This required a compression experiment with mouse skin, as well the use of a finite element model. As denoted in Appendix IV, the force relaxation predicted by the FE model well agrees with experimental data, with an average *R* ^2^ = 0.932.

We found that as thickness decreases, residual stress ratio decreases, which means the skin relaxes to a greater extent. This finding agrees with a study by Escoffier et. al [16], who reported that relaxation time decreases as people age, and we know that skin thickness decreases with aging [15]. Also, we identified that the residual stress ratio decreases with lower levels of stretch, i.e., higher strain levels, which echoes Funk et. al [21] who reported the same effect in ankle ligaments. The work herein is the first to report a decrease in time constant under a faster strain rate from biological measurements.

Although the dependency of the skin’s mechanical properties on strain and strain-rate is constitutively defined as material non-linearity, the dependency on skin thickness indicates that skin specimens of varying thickness are essentially different materials. Additional analyses indicate that the dependency of skin viscoelasticity on thickness and strain level are neither from frictional edge effects (computational finite element analysis, Appendix II) nor from different dermis/epidermis thickness ratios (statistical regression, Appendix III).

**Fig 8:**
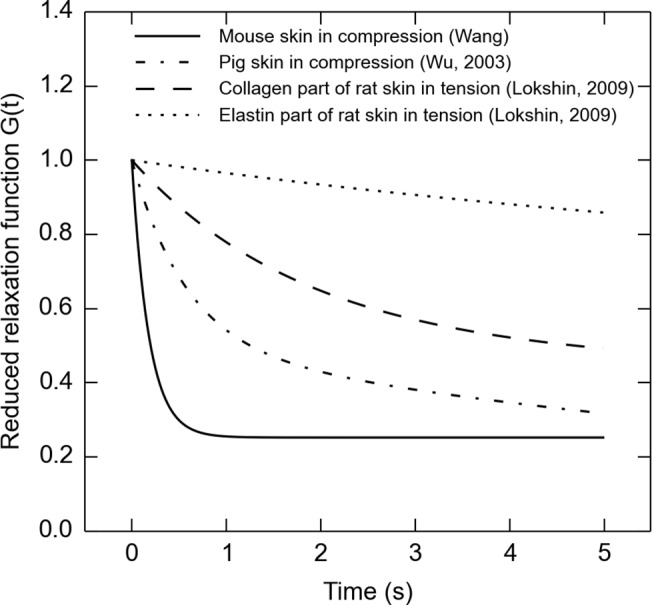
Comparison of the reduced relaxation function (Equation 5) from measurement of different skin samples. The solid line shows median data from work presented here on mouse hindlimb skin fitted to the one-term model, in the first experiment with median strain rate $\dot{\varepsilon }=3.54\;{\text{s}}^{-1}$. The dotted-dash line gives a measurement from pig dorsal skin [11]. The dashed and dotted lines are both from rat skin, but the dashed line function is attributable only to collagen elements in the skin while the dotted function is only elastin elements [4]. Note that the skin in compression relaxes more than skin in tension.

Our results from mouse hindlimb skin are comparable to prior tests of compression with pig dorsal skin [11], exhibiting similar time constants within a 5-second time-scale (median *τ*_1_ = 0.18 s from our one-term model fit compared to *τ*_1_ = 0.57 s on pig skin) and residual stress ratios (median *G*_*∞*_ = 0.284 herein, compared to *G*_*∞*_ = 0.234 on pig skin). However, if we compare the reduced relaxation functions of skin under compression with those of rat skin under tension [4], the compression curves are clearly distinguishable by their significantly smaller residual stress ratio *G*_*∞*_ (Fig. 8). Another key difference compared with that prior work is our use of skin from the hindimb, instead of dorsal skin, which is more commonly measured. The measurement of hindlimb skin is vital for studies of the sense of touch [26], known to be dependent on skin mechanical properties [18]. In particular, slowly adaptive type I (SAI) mechanosensitive afferents, essential for our ability to discriminate edges and curvature [30], display firing rate decay under constant displacement stimuli. This phenomena is known as adaptation and is dependent, in part, on the skin’s viscoelastic relaxation [31]. We chose a hold phase at the maximum load position of 5 seconds to align with such adaptation and the typical length of neurophysiological recordings from SAI afferents [26]. Therefore, one would need to be careful in extrapolating the conclusions of this work outside of the chosen time window.

Furthermore, the results of this work give important insights into issues currently being examined in the field of tactile mechanotransduction. SAI adaptation may carry information about a mechanical stimulus, for example, an object’s compliance. Since thinner skin relaxes more than thicker skin, these data predict that the neural response from a population of SAI afferents in thin skin might adapt their firing rates to a greater extent than a similar population in thicker skin. This could negatively affect the ability of those with thin skin (e.g., the elderly population) to accurate assess tactile stimuli. In concordance with this, it is known that tactile acuity decreases with age [32]. Studies investigating changes in tactile sensation with aging or after injury usually focus on neuronal causes, but our results suggest skin mechanics might also contribute to changes in tactile sensation. Our understanding of such mechanical properties—at the level of macro-scale compression—is important to develop realistic models of touch stimuli for haptic technology [18].

The results presented herein are based the assumption of a spatially homogeneous constitutive model; however, the skin is a heterogeneous and anisotropic material, and it is yet unclear what microscopic mechanisms underlie the nonlinear viscoelasticity we observe at the bulk level. Sub-micron studies have begun to suggest that individual skin layers indeed exhibit different degrees of viscoelasticity [9]. This may indicate that viscoelastic nonlinearity at the bulk level are dominated by one or more specific layers, such as the dermis, or a specific constituent, such as the interstitial fluid.

It is worth noting that there are some anatomical differences among various types of skin. The structure of skin differs between mouse hairy skin, our testing site, and glabrous skin. Hairy skin is composed of a thin epidermis that involutes deep into the dermis to form hair follicles. By contrast, glabrous skin, which lacks hair follicles, has a thick epidermis with undulating ridges at the dermal-epidermal junction. Human skin comprises the same fundamental layers as mouse skin with different thickness for each layer, with the exception that the muscular layer of panniculus carnosus in mouse skin does not exist in most areas of human skin [33]. In both species and both types of skin, the density and structure of the layers changes over the course of an animal’s life, as the dermal papillary ridges flatten with age [34] and hair follicles undergo growth cycles [35]. While we have standard testing data for murine skin [4,12], the existing literature on human specimens covers only in-vivo viscoelastic measurement with complex stress fields, for example, Krueger et al. [13] investigated how viscoelasticity changes with aging using a Cutometer. Future work on human skin specimens are needed to provide hyper-viscoelastic constitutive parameters, and the contribution of each layer to the skin’s viscoelastic nonlinearities and the changes in these properties with age is yet to discover for both species, in order to be used for numerical simulations to better aid clinical practice.

Our work suggests that normal features of the neuronal response could be mediated by skin mechanics. In particular, we hypothesize that SAI afferents may adapt their firing rates more quickly to strong stimuli than to weak stimuli, since the skin relaxes more under high-strain conditions. Such changes in neuronal firing could be one mechanism by which the nervous system gains information about stimulus properties. Furthermore, SAI afferents may adapt their firing rates more quickly to faster stimuli than to slower stimuli, since the skin relaxes more quickly under higher strain rates [31,36]. This said, one must also note that intrinsic neuronal properties play a role in the overall adaptation of the mechanosensitive response independent of the skin’s response. These results suggest a need to carefully control stimulus magnitude and velocity in performing electrophysiology experiments with tactile stimuli [18].

### Appendix I: Details of constitutive model selection and numerical implementation

**Fig 9:**
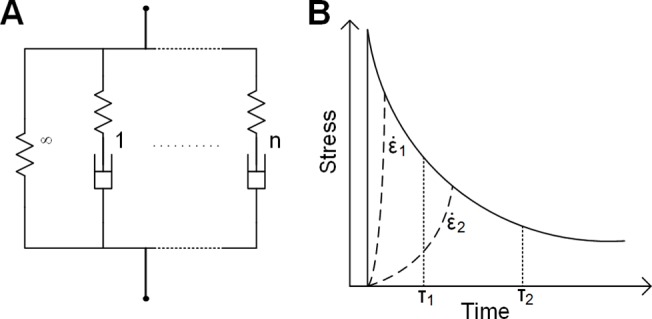
Schematic and illustration of the constitutive model. A: Rheological representation of the viscoelastic model, where the material consists of parallel chains, and each chain consists of an elastic component (denoted by a spring) and a viscous component (denoted by a dashpot). Usually, the steady-state response of a viscoelastic solid is represented by a chain with no viscous component (i.e., *τ* = ∞). Here, in addition to one solid chain, models including one and two chains with viscous components are evaluated and denoted as one-term and two-term models. B: Illustration of how stimuli with a low strain-rate may lose information from low time-constant QLV chains. The solid line is the response of a typical two-term viscoelastic solid with time constants *τ*_1_ and *τ*_2_ under a step load. Two dashed lines represent the response of the material under slower strain-rates. For the slowest strain-rate, ${\dot{\varepsilon }}_{2}$, stress relaxation properties of the faster chain (*τ*_1_) may not show up because its relaxation for the faster chain takes place within its ramp phase. Therefore, this will not be captured by curve fitting, which simply characterizes the material as a one-term QLV solid and only calculates the slower chain (with time constant *τ*_2_). Thus, we can eliminate the two extra parameters (*G*_1_, *τ*_1_) if we only care about low strain-rate situations. In other words, for low strain-rate cases, the single term model is sufficient and therefore more appropriate than the two-term models because of the reduced number of free parameters.

Hyper- and visco-elastic models have been adopted to fit the behavior of the experimental data, as previous efforts [11] have shown that skin under compression is hyper-viscoelastic. On a macro-scale, most biological tissues are viscoelastic [24] and have well-developed material models depending on the deformation level. Under small deformation, various spring-dashpot models have been used, including the most commonly used Kelvin-Voigt model, a standard linear solid model and generalized Maxwell model (i.e., Maxwell-Wiechert model) [37]. As biological tissue often undergoes finite deformation, these linear models must be modified to incorporate hyperelastic components. Two of the most popular models are the quasi-linear viscoelastic (QLV) model [24] and parallel-network viscoelastic (PNV) model [38]. Although the PNV model yields accurate and stable strain-energy outputs, the QLV model is more popular because the parameters are typically easier to interpret and it has a longer history (Fig. 9).

For the QLV model, a convolution integral was used to calculate stress from strain data,

$$\sigma \left(t\right)=\overset{t}{\underset{-\infty }{\int }}G\left(t-t'\right)\frac{\partial \sigma_{e}\left(\lambda \right)}{\partial \lambda }\frac{\partial \lambda \left(t^{\prime }\right)}{\partial t^{\prime }}dt'\;,$$ (3)

where *t* and *λ* denote time and stretch. The instantaneous elastic function of material is *σ*_*e*_ (*λ*), where herein we utilized a 1^st^-order Ogden form of the hyperelastic strain energy function [39],

$$\sigma_{e}\left(\lambda \right)=\frac{2\mu }{\alpha }\left(\lambda^{\alpha }-\lambda^{-\frac{\alpha }{2}}\right),$$ (4)

with *μ* and *α* being the material constants, *μ* also known as instantaneous elastic modulus. *G*(*t*) is defined as the reduced relaxation function, and is in the form of a Prony series,

$$G\left(t\right)=\overset{n}{\underset{i=1}{\sum }}G_{i}e^{-\frac{t}{\tau_{i}}}+G_{\infty }\;,$$ (5)

where *τ*_*i*_ were the time constants associated with stress relaxation ratio *G*_*i*_, and *G*_*∞*_ was the residual stress ratio at the steady state. At time *t* = 0 the value of *G*(*t*) was defined as unity,

$$\overset{n}{\underset{i=1}{\sum }}G_{i}+G_{\infty }=1\;.$$ (6)

The QLV models presented here include an Ogden elastic representation and a Prony series relaxation function utilizing one and two terms, (where *n* = 1 and *n* = 2 specifically), that were referred to as “one-term QLV” and “two-term QLV”, respectively. There are 2 independent viscoelastic parameters for the one-term model (*τ*_1_, *G*_*∞*_; note that *G*_1_ = 1 − *G*_*∞*_ and therefore is not independent) and 4 for the two-term model (*τ*_1_, *τ*_2_, *G*_1_, *G*_*∞*_) as shown in Equation (7) and (8), not counting the two hyperelastic parameters (*μ*, *α*). Since the one-term model had fewer free variables (at the cost of inability to predict response for high strain-rates, Fig. 9B) it was therefore preferred given similar goodness of fit to the two-term model.

$$G\left(t\right)=G_{1}e^{-\frac{t}{\tau_{1}}}+G_{\infty }\;,$$ (7)

$$G\left(t\right)=G_{1}e^{-\frac{t}{\tau_{1}}}+G_{2}e^{-\frac{t}{\tau_{2}}}+G_{\infty }\;,$$ (8)

In contrast to the previous recursive method [40], the implemented numerical algorithm is designed to be in vector form so for-loops can be avoided, thus it is much easier to implement in numerical packages like MATLAB and NumPy. We first start from Equation (3), which can be split by applying Equation (5):

$$\sigma \left(t\right)=\overset{n}{\underset{i=1}{\sum }}\sigma_{i}\left(t\right)+\sigma_{\infty }\left(t\right)\;,$$ (9)

Where

$$\sigma_{i}\left(t\right)=\overset{t}{\underset{-\infty }{\int }}G_{i}e^{-\frac{t-t^{\prime }}{\tau_{i}}}\frac{\partial \sigma_{e}\left(\lambda \right)}{\partial \lambda }\frac{\partial \lambda \left(t^{\prime }\right)}{\partial t^{\prime }}dt'\;,$$ (10)

$$\sigma_{\infty }\left(t\right)=\overset{t}{\underset{-\infty }{\int }}G_{\infty }\frac{\partial \sigma_{e}\left(\lambda \right)}{\partial \lambda }\frac{\partial \lambda \left(t^{\prime }\right)}{\partial t^{\prime }}dt^{\prime }\;,$$ (11)

By assuming no stress history before *t* = 0 and identifying the constants in Equation (10), we have

$$\sigma_{i}\left(t\right)=G_{i}e^{-\frac{t}{\tau_{i}}}\overset{t}{\underset{0}{\int }}e^{\frac{t^{\prime }}{\tau_{i}}}\frac{\partial \sigma_{e}\left(\lambda \right)}{\partial \lambda }\frac{\partial \lambda \left(t^{\prime }\right)}{\partial t^{\prime }}dt'\;.$$ (12)

And this is now ready for numerical implementation, as

$$\sigma_{i}^{k}=G_{i}e^{-\frac{t^{k}}{\tau_{i}}}\overset{k}{\underset{l=2}{\sum }}e^{\frac{t^{l}}{\tau_{i}}}\left(\sigma_{e}^{l}-\sigma_{e}^{l-1}\right)\;,$$ (13)

where the superscript *k* means the value at k^th^ point in time, i.e., *σ* ^*k*^ means stress at time *t* ^*k*^. The summation starts from *l* = 2 because we assert the stress change is zero at time zero. Also, from Equation (4) we calculate instantaneous stress as

$$\sigma_{e}^{k}=\frac{2\mu }{\alpha }\left[{\left(\lambda^{k}\right)}^{\alpha }-{\left(\lambda^{k}\right)}^{-\frac{\alpha }{2}}\right]\;,$$ (14)

Similarly, we can obtain

$$\sigma_{\infty }^{k}=G_{\infty }\overset{k}{\underset{l=2}{\sum }}\left(\sigma_{e}^{l}-\sigma_{e}^{l-1}\right)\;.$$ (15)

And the final stress evolution can be computed from

$$\sigma^{k}=\overset{n}{\underset{i=1}{\sum }}\sigma_{i}^{k}+\sigma_{\infty }^{k}\;,$$ (16)

Thus Equation (13–16) completes the numerical implementation of QLV model.

### Appendix II: Finite element analysis

To exclude the case where the frictional boundary conditions might confound the trend between thickness, stretch levels and viscoelasticity, numerical experiments of finite element (FE) analysis were performed using the commercial FE software package ABAQUS Standard, version 6.12 (Dassault Systèmes, Vélizy-Villacoublay, France).

**Fig 10:**
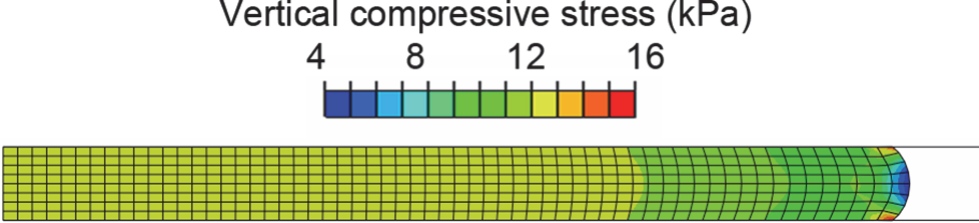
Typical distribution of vertical compressive stress (S22 in ABAQUS) from the axisymmetric FE simulation (therefore only the right half of the skin middle-section is shown), with friction coefficient of 0.3 and skin thickness of 400 μm. Note that there is only minor edge effect around the periphery.

Numerical experiments of skin specimens were performed using axisymmetric models (Fig. 10), in which the geometry was the same as the biological specimen (6 mm dia. cylindrical skin piece). The material parameters assigned were from Table 1, using the two-term QLV model for higher accuracy. Three frictional coefficients (*μ*_*f*_) between the skin and compression platen/table were tested, namely 0 (frictionless), 0.3 (between human finger and metal tip [41]), and ∞ (rough). The rough friction coefficient also accounts for the cohesive force between skin and the metal, given that our boundary condition enforces no separation after contact [42]. Contact behaviors were defined in both a) tangential behavior, where isotropic friction was specified with penalty friction formulation, and b) normal behavior, where “hard” contact pressure-overclosure was used. A high Poisson’s ratio *ν* = 0.475 for the skin were used. First, in line with the skin compression experiments, models of skin thickness from 200 to 800 μm with 100 μm increments was constructed. CAX4RH elements 50 μm in edge length were used. The stretch level and ramp time were both derived from the median values in the compression experiment where skin thickness varied, namely *λ*_*∞*_ = 0.63, *t*_*ramp*_ = 0.129 s. The aforementioned model with skin thickness of 400 μm was then modified for a second experiment on variability in stretch level, where steady-state stretches were varied from 0.2 to 0.8 with an increment of 0.1. After the analyses were completed, the reaction force and displacement at the compression platen were extracted and processed in the same manner as the data from the skin compression experiments (described in Section 2.6), and the viscoelastic parameters were then compared to those obtained from the compression experiments.

**Fig 11:**
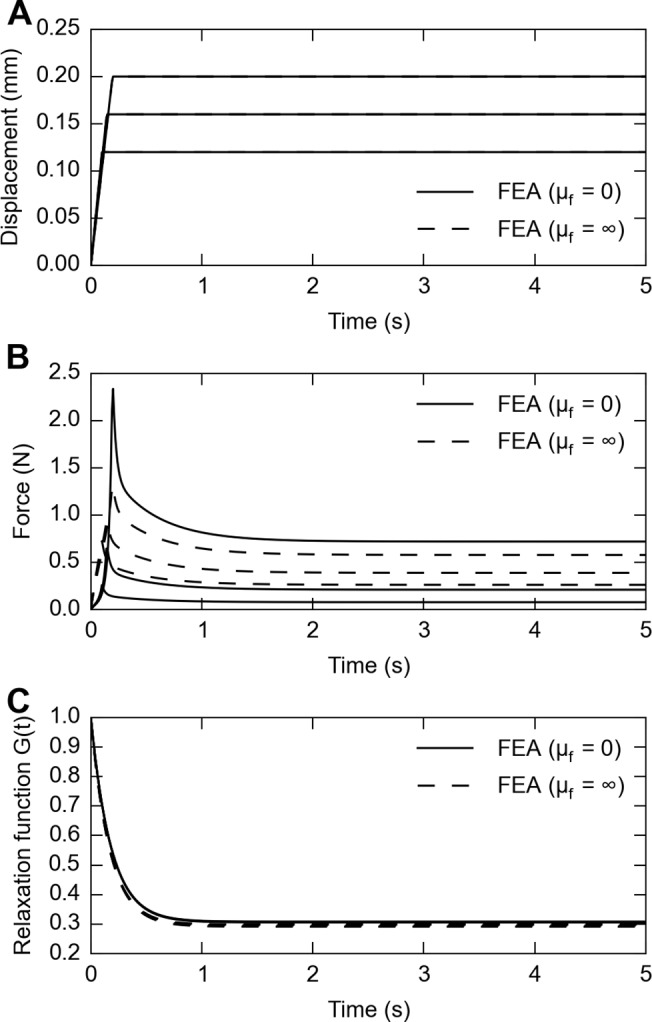
FE simulations for a skin thickness of 400 μm, at stretch levels of 0.5, 0.6 and 0.7, under frictionless (solid lines) and rough (dashed lines) friction conditions. A: displacement stimuli to achieve desired stretch level; B: responsive force traces for three stretch levels under different frictional conditions; C: calculated relaxation function for force traces shown in B. Note that while frictional conditions have an impact on responsive force traces, they do not impact the calculated viscoelastic reduced relaxation function.

**Fig 12:**
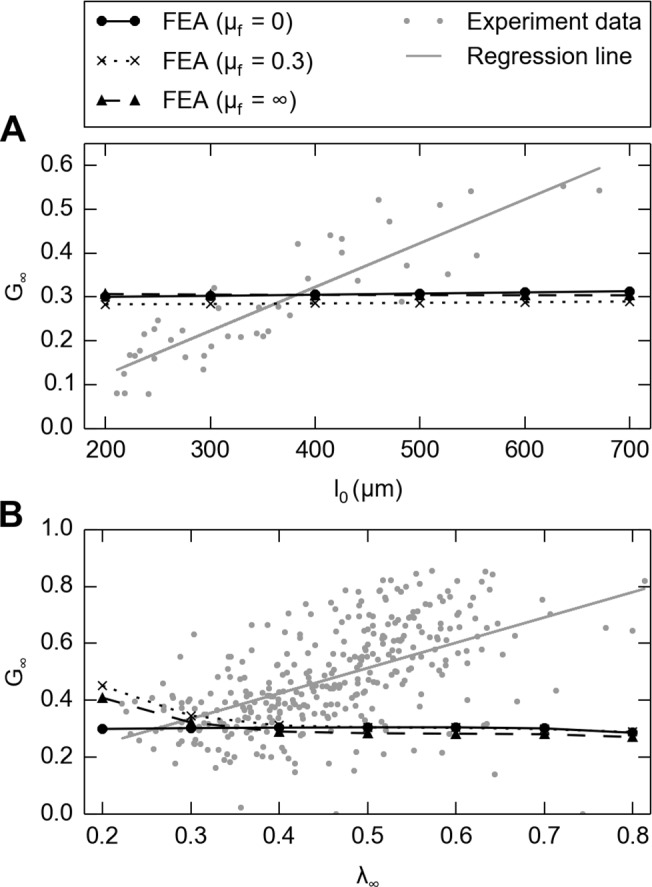
FE analysis shows minimal frictional edge effects on the calculated residual stress ratio, A: when skin thickness changes, plotted on top of Fig. 6A; and B: when strain level changes, plotted on top of Fig. 6B.

In Fig. 11 we showed FE simulations on same skin thickness (400 μm) but under extreme frictional conditions (frictionless and rough), for different stimulus magnitudes (*λ*_*∞*_ from 0.5 to 0.7). Reduced relaxation functions obtained from fitting the hyper-viscoelastic constitutive model was also plotted in Fig. 11C. This shows that although changes in frictional conditions result in different force responses, the viscoelastic reduced relaxation functions are not impacted. The final outcome of the FE analysis, testing whether the effect of thickness on viscoelasticity is caused by frictional edge effects or the innate property of the skin, showed that the edge effect negligibly influences the outcome (Fig. 12A), independent of three levels of friction coefficients. Similarly, the frictional edge effects negligibly influence the outcome caused by strain level as well (Fig. 12B).

### Appendix III: Role of dermis/epidermis thickness ratio

In addition to the absolute thickness of a skin specimen, another independent variable that might contribute to viscoelastic variability is the relative thickness ratios between skin layers, as this changes between skin sites of Distal, OffNT and NT. The thickness ratios between dermis and epidermis, as previously obtained for different skin sites [12], were used as the independent variable here. Value of this dermis/epidermis thickness ratio, denoted as *r*, is listed in Table 2.

**Table 2:** Value of dermis/epidermis thickness ratio *r* previously measured [12].

	Epidermis			Dermis		
	Distal	OffNT	NT	Distal	OffNT	NT
Average thickness (pixels)	16.30	15.00	11.70	194.00	352.80	288.50
	13.70	14.00	13.50	228.30	239.80	378.00
	16.30	14.30	14.20	195.80	259.90	405.00
	15.80	14.20	13.70	311.80	563.20	471.80
	15.30	14.00	13.70	260.20	575.80	567.20
	17.80	15.80	13.00	308.50	555.20	575.50
	15.80	13.50	14.80	150.20	256.80	634.30
	16.80	13.80	15.70	126.80	216.50	478.80
	15.50	13.20	14.50	128.00	257.50	496.30
Mean	15.92	14.20	13.87	211.51	364.17	477.27
Std.	1.12	0.79	1.14	71.22	154.95	108.11
n	9	9	9	9	9	9
				Distal	OffNT	NT
Epidermis/dermis thickness ratio				13.28	25.65	34.42

During the analysis of the first experiment when skin thickness varied, a multi-linear regression was performed with residual stress ratio *G*_*∞*_ as the dependent variable, and returned *p* < 0.001 for the independent variable thickness *l*_0_ but *p* = 0.63 > 0.05 for the independent variable dermis/epidermis thickness ratio *r* (Table 2). This indicates that the impact for the dermis/epidermis thickness ratio *r* was insignificant on residual stress ratio.

### Appendix IV: Experiment to validate the viscoelastic material model in a secondary context

**Fig 13:**
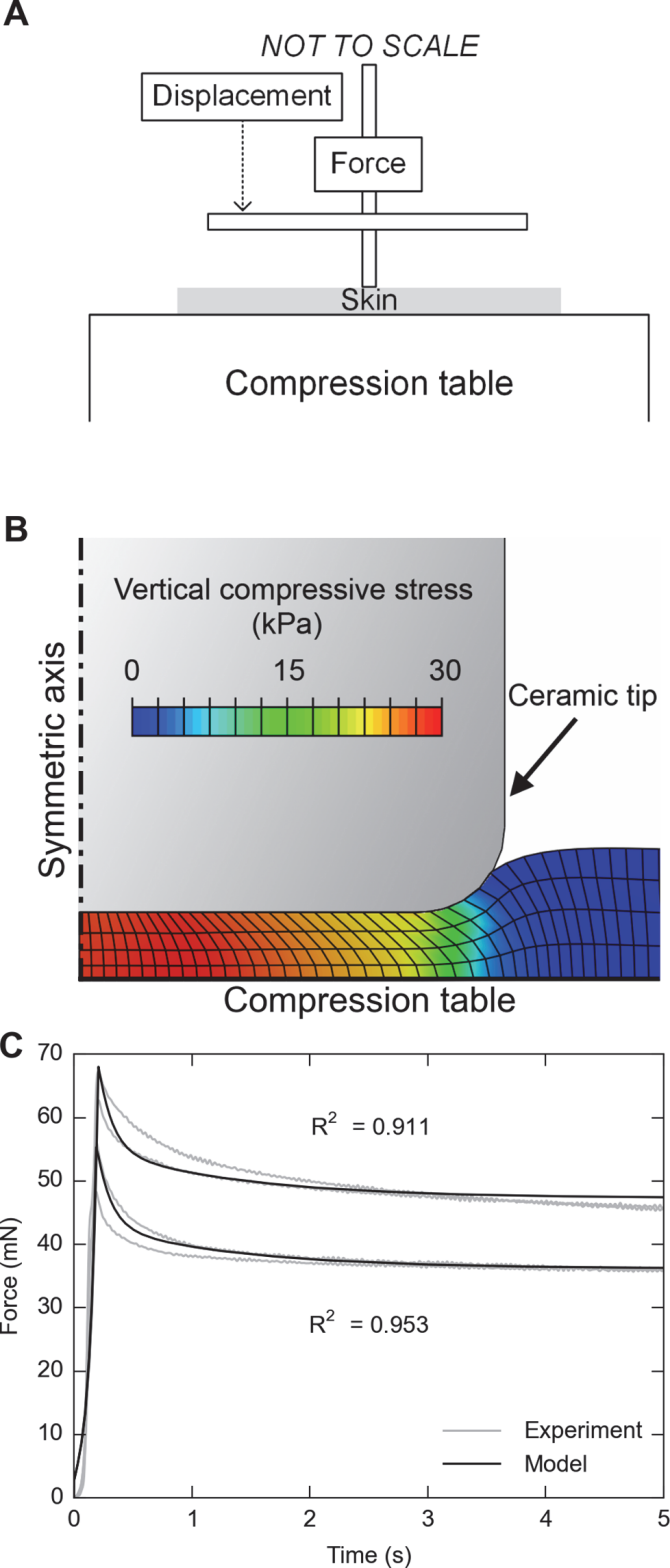
Validation experiment in a secondary context, using a 1.5 mm diameter tip and 8 mm diameter skin specimen, to demonstrate the applicability of the measured QLV parameters. A: Schematic drawing of the experimental set-up; B: Finite element model with the contact region magnified; C: FE analysis shows good agreement between numerical prediction and actual experimental measurement.

An additional compression experiment with mouse skin was performed with a small ceramic tip of 1.5 mm diameter, using a larger skin specimen of 8 mm diameter, sampled from the right hind leg of a 9.4-week-old mouse. The skin specimen was placed on the aforementioned aluminum compression table with sufficient SIF to prevent skin from drying (Fig. 13A). Four ramp and hold displacement-controlled indentations were commanded at 1 mm/s, i.e., two iterations at two magnitudes, which were chosen to achieve approximately *λ*_*∞*_ = 0.5. Force-time data were recorded, from which probe-to-skin contact points were determined when force crossed a threshold of 0.5 mN. The data were post-processed with Python packages of SciPy and NumPy.

To predict the result of this experiment using material model data from the flat plate experiments, a finite element analysis was performed in ABAQUS Standard. The model was constructed of approximately 800 CAX4RH elements (Fig. 13B). The skin thickness was 225 μm, which is at the median for a 9-week-old mouse [12]. Four layers of equal-sized elements were specified in the thickness direction. One hundred single-biased elements were specified in the radial direction with a bias ratio of 5 and higher mesh density near the symmetric axis than the peripheral axis. The ceramic tip was modeled as 0.75 mm radius cylinder with fillet radius of 0.15 mm. The friction coefficient between the ceramic and skin was chosen as 0.3 [43], and 0 between the aluminum and skin. For all contact interactions, “hard” contact, pressure overclosures were used and no separation after contact was allowed. Material properties from the second experiment with the flat plate indenter were used, which employed the two-term model (4^th^ line in Table 1).

Good agreement was observed between the force-time curves from FE model prediction and from the experiment, with an average *R* ^2^ = 0.932 (Fig. 13C). A rapid decay at the early relaxation (< 1 s) was followed by one more gradual (1–5 s) and observed in both the experiment and the model prediction. The accurate prediction in this new context—given changes in skin size, indenter tip, and boundary conditions—demonstrates the applicability of QLV constitutive parameters presented herein, within the time window between 0–5 s.
